# Diffuse non-obstructive coronary artery disease: two clinical faces of the same disease—a case report

**DOI:** 10.1093/ehjcr/ytad605

**Published:** 2023-12-22

**Authors:** Matjaž Klemenc, Gregor Budihna, Igor Kranjec

**Affiliations:** Department of Intensive Care Unit, General Hospital of Nova Gorica, Padlih borcev 13A, Šempeter pri Gorici 5290, Slovenia; Department of Intensive Care Unit, General Hospital of Nova Gorica, Padlih borcev 13A, Šempeter pri Gorici 5290, Slovenia; Department of Cardiology, University Medical Centre, Ljubljana, Slovenia

**Keywords:** ST-segment elevation myocardial infarction, Effort angina, MINOCA, Diffuse coronary atherosclerosis, Case report

## Abstract

**Background:**

Coronary artery disease has a long preclinical phase before manifesting itself clinically due to diffuse non-obstructive disease, stenoses, or thrombosis.

**Case summary:**

We present a case of a middle-aged male complaining of atypical chest pain, then severe retrosternal pain, and, eventually, effort angina. We performed non-invasive testing, coronary angiography, intravascular imaging, and flow reserve tests, each as appropriate. Cardiovascular risk control, optimization of drug therapy, and percutaneous coronary intervention were considered trying to comply with the best clinical practice.

**Discussion:**

Diffuse non-obstructive coronary artery disease may present clinically in different ways. Exercise stress test might be sufficient to assess effort angina before a potential angiography. Flow reserve tests across the diseased vessel can distinguish between diffuse and focal pattern of the disease and assist in the adequate selection of therapy. Finally, intravascular imaging is invaluable for the assessment of the plaque risk features.

**Learning points**

- Diffuse non-obstructing coronary artery disease may present clinically in different ways.
- Flow reserve tests, including pullback across the diseased vessel, can distinguish between diffuse and focal pattern, and intravascular imaging may find the pathologic hallmark of the acute coronary syndrome.
- Appropriate selection of the treatment modalities depends critically on the findings of the invasive tests.

## Introduction

Coronary artery disease (CAD) is a pathological process characterized by atherosclerotic plaque accumulation in the epicardial arteries, whether obstructive or non-obstructive. In the Swedish Cardiopulmonary Bioimage Study, any coronary atherosclerosis was common (42.1%), while significant stenoses (≥50%) were limited (5.2%) in a random sample of a middle-aged population examined with coronary computed tomography.^1^ Coronary artery disease has a long preclinical phase before manifesting itself clinically due to diffuse non-obstructive disease, stenosis, or thrombosis. The dynamic nature of CAD results in various presentations, which can be conveniently categorized as either acute or chronic coronary syndrome. Data from the Western Denmark Heart Registry showed that plaque burden and not stenosis by itself was the main predictor of serious cardiovascular events.^2^

We report herein a case of diffuse non-obstructive CAD, resulting successively in both ST-segment elevation myocardial infarction (STEMI) and effort angina.

## Summary figure

**Figure:**
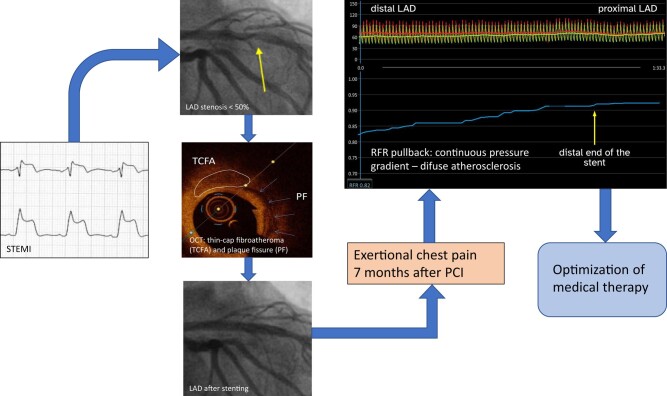


## Case presentation

**Figure 1:**
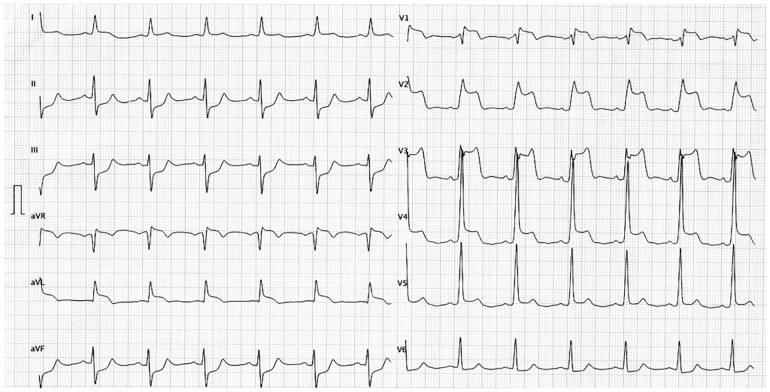
Electrocardiogram (ECG) at first medical contact. Pronounced ST-segment elevations are seen in the leads I, aVL, and V_1–4_.

A 62-year-old male with several risk factors (hypertension, hypercholesterolaemia, and smoking) complained of occasional chest discomfort for a few months. Finally, he was referred to the emergency department for evaluation of severe retrosternal pain accompanied with shortness of breath. His physical examination was unremarkable (blood pressure 149/99 Hg, heart rate 69/min, saturation of arterial blood without added oxygen was 97%), but electrocardiogram revealed pronounced ST-segment elevations in anterior leads (*Figure 1*).

**Figure 2:**
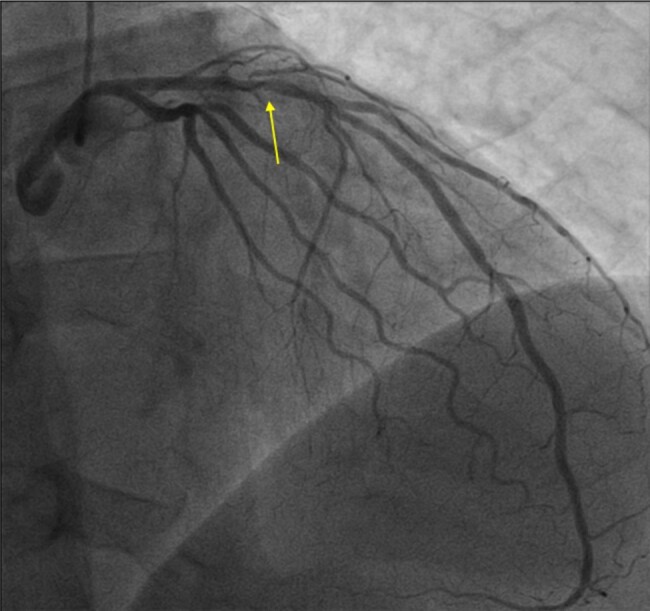
Initial left coronary angiogram in cranial right anterior oblique (c-RAO) view. The culprit lesion (arrow) is moderate and distal blood flow is preserved.

**Figure 3:**
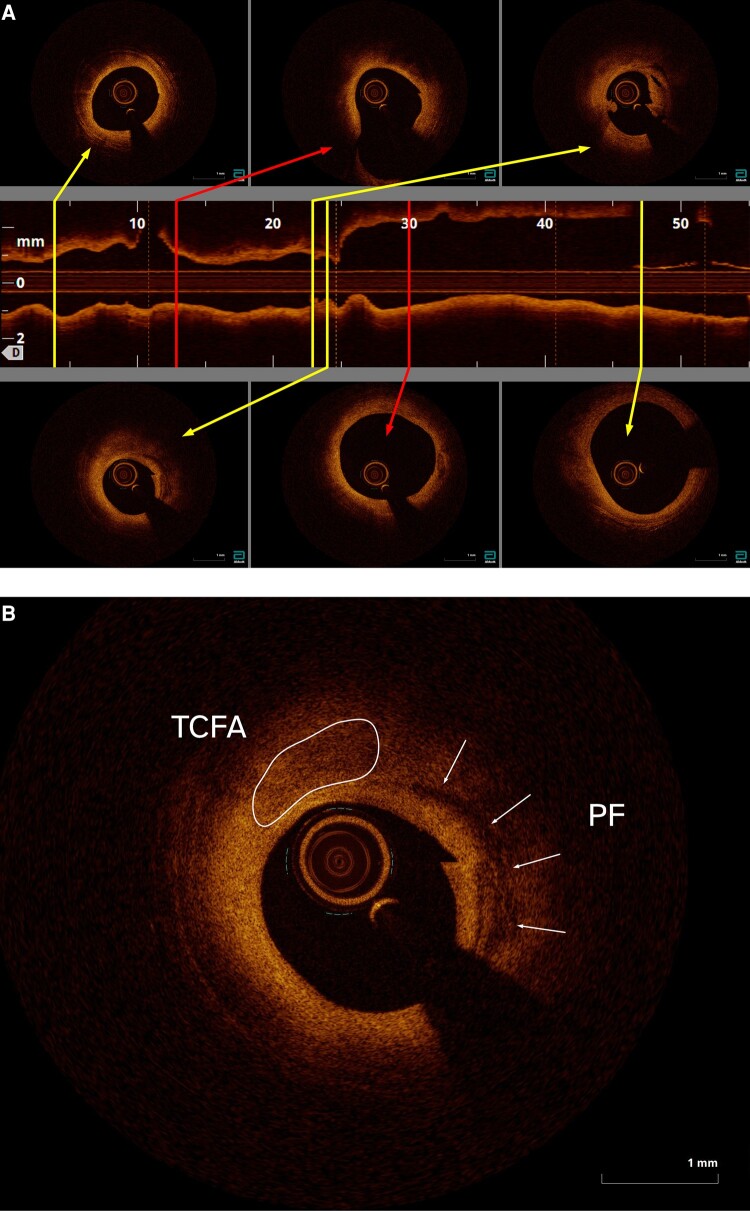
(*A*) Initial optical coherence tomography (OCT) pullback through the left anterior descending artery (LAD). The distal part of the artery is on the left side and the proximal part on the right. The culprit lesion is delimited with two red lines; it contains thin-cap fibroatheroma (TCFA), plaque fissure (PF) (first image in the lower panel), and superimposed white thrombus (third image in the upper panel). (*B*) Mid-culprit vessel site. Thin-cap fibroatheroma is seen from 10 to 12 o’clock and extends into the plaque fissure.

He was immediately taken to catheterization laboratory. Angiography suggested a diffuse CAD; surprisingly, the left anterior descending artery (LAD)—a presumed culprit—showed only a moderate obstruction of the mid-vessel with well-preserved blood flow (*Figure 2*, Supplementary material online, *Videos S1*–*S6*). Optical coherence tomography (OCT) was performed to address disparities between electrocardiographic and angiographic findings. The impression of diffuse CAD was confirmed since no ‘healthy’ arterial structure was seen within the examined LAD (*Figure 3A*); indeed, the intimal thickness throughout the vessel exceeded 600 µm. The massive plaque burden comprised mostly fibrous plaques. The culprit lesion with a minimal diameter 1.77 mm, minimal luminal area 2.46 mm^2^, per cent diameter stenosis 44%, and per cent area stenosis 66% appeared in the mid-LAD. Importantly, the hallmark of an unstable lesion such as a thin-cap fibroatheroma, plaque rupture, and thrombus was clearly seen (*Figure 3B*).

Having recognized the high-risk features of the culprit, a decision was made to proceed with percutaneous coronary intervention (PCI) and a 2.75 × 22 mm drug-eluting stent was placed across the lesion. Stent apposition was considered adequate, minor malapposition was corrected with a larger non-compliant balloon, no edge dissections were observed, and the blood flow remained normal.

The high-sensitivity troponin T peaked the same day at 27 ng [upper limit of normal (ULN), 14 ng/L), Q-waves did not develop, and myocardial contractility seemed unaffected (ejection fraction, 57%). The hospital course was uncomplicated, and he was discharged on daily doses of aspirin 100 mg, ticagrelor 180 mg, nebivolol 2.5 mg, perindopril 2 mg, and rosuvastatin 20 mg.

The patient resumed his previous activities, took medication regularly, and the low-density lipoprotein (LDL) cholesterol decreased to 1.4 mmol/L. However, the chest discomfort persisted while riding his bicycle. Typical symptoms were provoked at high exercise workload (13.5 metabolic equivalents) with ST-segment depressions (0.35 mV) in lateral precordial leads.

**Figure 4:**
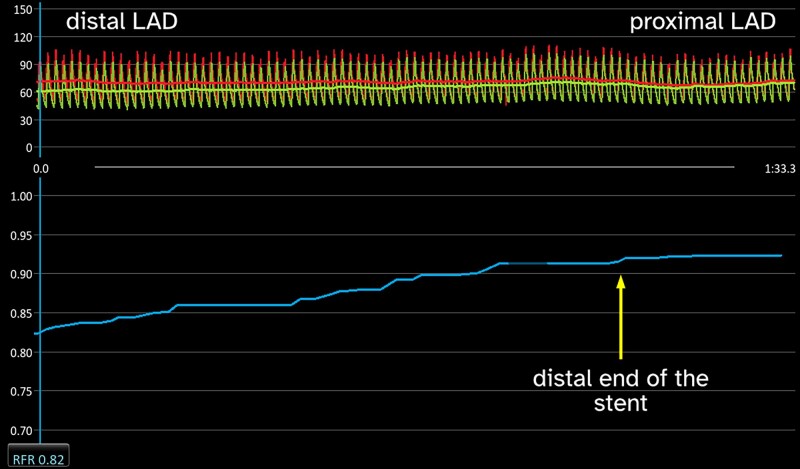
Resting full-cycle ratio (RFR) pullback through the left anterior descending artery (LAD) 7 months after the primary event. The distal part of the artery is on the left side and the proximal on the right. A continuous pressure gradient representative for the diffuse atherosclerosis is observed throughout the entire LAD; there is no significant step-up at the stent site confirmed by simultaneous angiography.

He was readmitted to the hospital for invasive coronary evaluation. On angiography, the stented segment remained patent and there was no progression at other coronary sites. However, fractional flow reserve (FFR) after 200 µg intracoronary adenosine was impaired in LAD [0.72, lower limit of normal (LLN), 0.80] as compared with the left circumflex artery (0.96). Moreover, the resting full-cycle ratio (RFR) was decreased to 0.82 (LLN, 0.90); the pullback from the distal LAD showed a continuous gradient without a step-up at the stent level (*Figure 4*). The procedure was terminated without further intervention.

The patient was instructed to pursue his physical activities and carry on with his previous medications along with ranolazine 325 mg b.i.d. and trimetazidine 35 mg b.i.d. Three months later, his clinical condition remained stable.

## Discussion

A seemingly straightforward case of STEMI raises several interesting issues, mainly of myocardial infarction with non-obstructive coronary arteries (MINOCA) and diffuse CAD without focal obstructions.

ST-segment elevation myocardial infarction is usually caused by the occlusion of the major epicardial artery provoked by the rupture/erosion of the vulnerable plaque. Cyclic flow variations are typical for the culprit arteries with progressive flow reduction and its rapid restoration reflecting thrombus formation and dislodgment.^3^ It is difficult, however, to ascertain the severity of the pre-existing lesion before the acute event. Data indicate that about 80% of STEMI patients have culprit stenosis >50% following restoration of blood flow with thrombolysis;^4^ conversely, 10% of patients with acute coronary syndrome and 4% with STEMI do not have obstructive CAD.^5^

Myocardial infarction with non-obstructive coronary arteries requires that the usual criteria for acute infarction are met and no stenosis ≥50% in the major epicardial artery is detected. It is important to acknowledge the weakness of coronary angiography to assess lumen geometry, plaque components, and presence of thrombus.^6^ Indeed, plaque rupture has been identified using intravascular ultrasound in 40% of the MINOCA cases.^7^ Our case confirms that OCT can definitely identify the morphologic hallmark of STEMI including plaque rupture and thrombus.

Primary PCI has become treatment of choice for STEMI; however, dedicated working groups have not encouraged PCI for the MINOCA subset.^5^ The aggressive preventive therapy is, thus, the mainstay of recommended therapy. However, MINOCA is not a benign clinical entity: a 12-month all-cause mortality of 4.7% far exceeds comparative rates of patients with stable CAD.^8^ Moreover, 17% STEMI patients with spontaneous reperfusion experienced recurrent events requiring emergency PCI in 10.6%.^9^ In intermediate lesions, the reasonable criteria for performing PCI are minimal area <2.5 mm^2^, plaque rupture, and presence of thrombus.^2,10^ Finally, it has been speculated that PCI might induce sufficient intimal proliferation preventing the rupture plaque to occlude.^11^ Therefore, we decided to perform PCI on the culprit lesion with minimal area 2.46 mm^2^ and morphologic high-risk features.

Pathologists believed CAD to be a focal rather than a diffuse intimal disease. However, newer diagnostic tools such as intravascular imaging and blood flow reserve studies have confirmed the diffuse nature of CAD. In a recent study of pathophysiological patterns of CAD, only 50% of the diseased vessels were identified as focal, 22% as diffuse, and 28% as combined pattern.^12^ Functional assessment is crucial to manage the MINOCA patients. Non-hyperaemic indices have the ability to detect significant stenosis at an acceptable level when compared with FFR. Instantaneous wave-free ratio (iFR) and RFR are highly concordant and diagnostically equivalent, and the optimal cut-off values are the same.^13^ Diffuse CAD without focal obstructions causes a continuous decline in coronary pressure along the arterial length: it is defined as trans-stenotic pressure gradient <0.03 over the length >15 mm by iFR or <0.05 over the length >25 mm by RFR.^14^ Finally, diffuse CAD without focal obstructions can be a substrate for plaque rupture and consequent STEMI because of the greater number of lesions that are less frequently protected with collaterals.^15^

## Conclusion

Coronary artery disease has a long preclinical phase before manifesting itself clinically due to diffuse non-obstructive disease, stenoses, or thrombosis. Diffuse CAD may present with effort angina or myocardial infarction. In the first case, iFR/RFR pullback may be necessary, in the second intravascular imaging, possibly with OCT. Optimal medical therapy is the mainstay of management, though PCI might be useful in the high-risk culprit lesions.

## Supplementary Material

ytad605_Supplementary_Data

## Data Availability

The data underlying this article are available in the article and in its online Supplementary material.

## Lead author biography

Matjaž Klemenc, MD, PhD, FESC, is an interventional cardiologist, working in the General Hospital of Nova Gorica, Slovenia.

## Supplementary material

**Consent:** The authors confirm that written consent for submission and publication of this case report, including images and associated text, has been obtained from the patient in line with the Committee on Publication Ethics (COPE) guidance.

**Funding:** None declared.
